# Human bony labyrinth dataset: Co-registered CT and micro-CT images, surface models and anatomical landmarks

**DOI:** 10.1016/j.dib.2019.104782

**Published:** 2019-11-09

**Authors:** Wilhelm Wimmer, Lukas Anschuetz, Stefan Weder, Franca Wagner, Hervé Delingette, Marco Caversaccio

**Affiliations:** a Department of ENT, Head and Neck Surgery, Inselspital, Bern University Hospital, Switzerland; b Hearing Research Laboratory, ARTORG Center for Biomedical Engineering Research, University of Bern, Switzerland; c Université Côte d’Azur, Inria, Epione, Sophia Antipolis, France; d Department of Diagnostic and Interventional Neuroradiology, Inselspital, Bern University Hospital, Switzerland

**Keywords:** Cochlea, Vestibule, Semicircular canals, Inner ear, Morphology, Anatomy

## Abstract

The presented data set consists of images, labels and surface models of 23 human bony labyrinths. For each specimen clinical computed tomography (CT) and co-registered high-resolution micro-CT images were acquired. Using the images, the bony labyrinth was segmented and 3D surface models were generated. Each specimen is accompanied by a description file containing the coordinates of anatomical landmarks and the corresponding cochlear coordinate system. The data set can be used to study the morphology of the inner ear or to evaluate segmentation algorithm as used for the preoperative planning of surgical procedures such as cochlear implantation.

**Table:** Specifications Table

Subject	Otorhinolaryngology and Facial Plastic Surgery
Specific subject area	Inner ear anatomy
Type of data	Table Image Computed Tomography and Micro-computed tomography images
How data were acquired	Ex-Vivo specimen were imaged using computed tomography imaging and micro computed tomography imaging The data was labelled, segmented and 3D surfaces were generated
Data format	Raw Analyzed (Labelled) Co-Registered
Data source location	Institution: University of Bern City/Town/Region: Bern Country: Switzerland
Data accessibility	Repository name: Zenodo Data identification number: DOI/10.5281/zenodo.3355272 Direct URL to data: https://zenodo.org/record/3355272
Related research article	Authors: Wilhelm Wimmer, Clair Vandersteen, Nicolas Guevara, Marco Caversaccio, Hervé Delingette Title: Robust Cochlear Modiolar Axis Detection in CT Journal: MICCAI: International Conference on Medical Image Computing and Computer-Assisted Intervention, 978-3-030-32253-3, MICCAI 2019, Part V, LNCS 11768 DOI: 10.1007/978-3-030-32254-0_1

**Table:** 

**Value of the Data** • A high-qualitative, co-registered data set of the human bony labyrinth that can be used to study macroscopic inner ear morphology in detail. The raw imaging data, surface models and anatomical landmarks are provided. • In the fields of otology and neurotology, an application lies in the design of neuroprostheses, including cochlear and vestibular implants to provide minimally invasive surgical treatment of hearing and balance disorders. • Anthropologists could use reliable morphological descriptions to for analysis, since the shape of the bony labyrinth encodes information about the genetic distance from early humans as well as sex-typed differences. • The data can be used to design and test segmentation algorithms for surgical planning and postoperative radiological outcome evaluation. The co-registered high-resolution uCT data can serve as reference.

## 1 Data

**Fig. 1:**
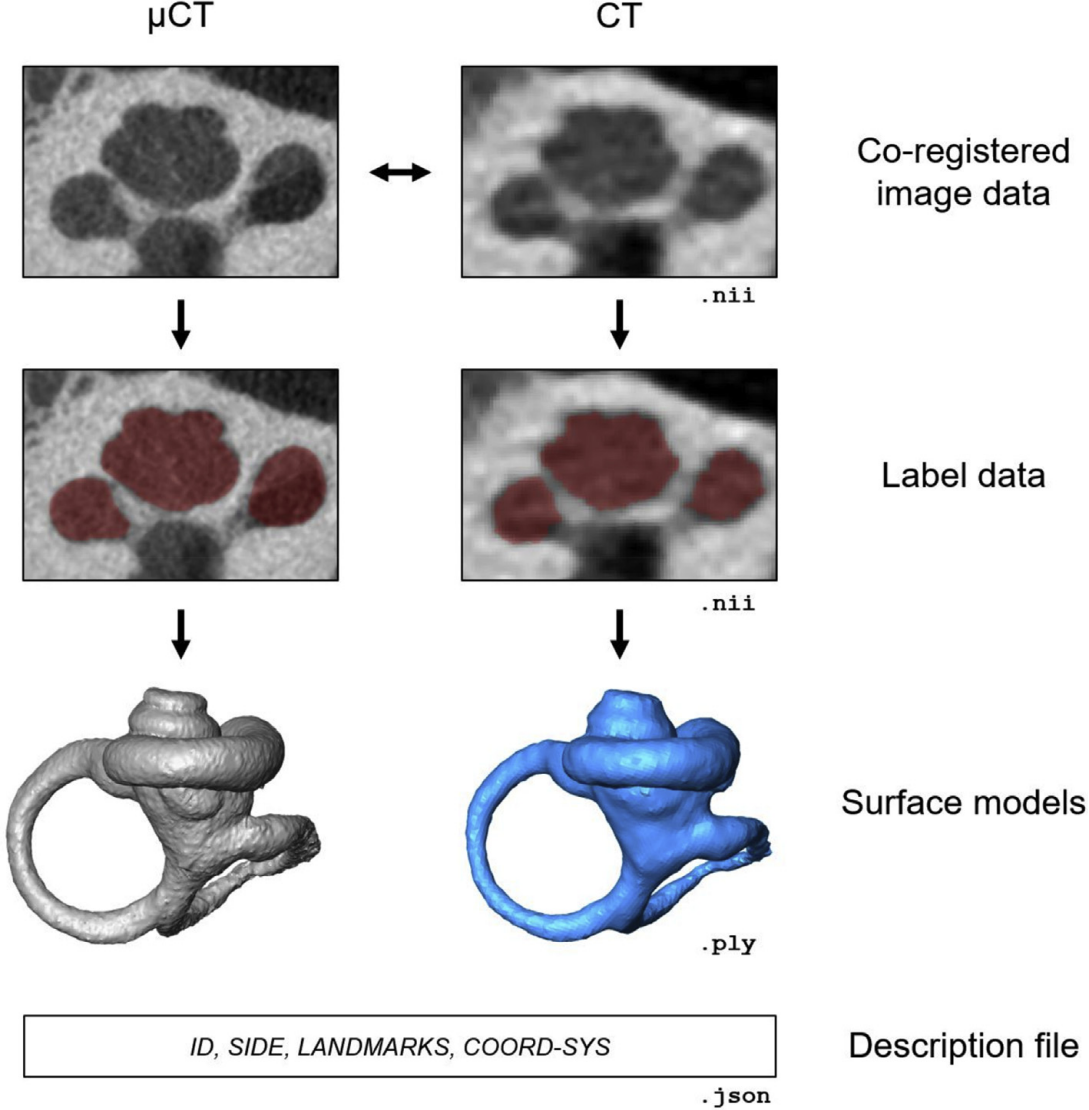
Overview of the data contained for each of the 23 specimens.

The data set comprises a total of 23 specimens and can be downloaded free of charge from the Zenodo open access data repository through a permanent Digital Object Identifier. The data of each specimen is packed in a single compressed file (.zip) containing two folders (CT and uCT) and one description file (see Fig. 1). A detailed description of the data items is given in Table 1. The description file contains specimen identifiers, the coordinates of anatomical landmarks in world coordinates and the corresponding cochlear coordinate system (Table 2). The information is stored in a text file using the JavaScript Object Notation (.json) format. Image and label data are saved as NIFTI (Neuroimaging Informatics Technology Initiative) files (.nii). Because of the co-registration, for a given specimen, all images and labels share the same coordinate system. In addition, each folder contains a triangulated surface model stored in the ASCII Stanford Triangle Format (.ply).

**Table 1:** Data content for each specimen (packed in a.zip-file). XXX denotes a placeholder for the corresponding specimen identification number, e.g. F01.

File	Description	Format
XXX_DESC	specimen description file (see Table 2)	. json
XXX_CT_RAW	CT image data	.nii
XXX_CT_LABELS	CT label data	.nii
XXX_CT_SURF	CT surface model data	.ply
XXX_uCT_RAW _CT	image data	.nii
XXX_uCT_LABELS _CT	label data	.nii
XXX_uCT_SURF _CT	surface model data	.ply

**Table 2:** Information stored in a specimen descriptor file.

Item	Description
ID	Identification number
SIDE	Ear side
RW	Landmark: center of the round window
C	Landmark: center of the cochlear basal turn
A	Landmark: helicotrema
OW	Landmark: center of the oval window
V	Landmark: center of the vestibule
ORIGIN	Coordinate system: origin
XAXIS	Coordinate system: x unit vector (towards round window)
YAXIS	Coordinate system: y unit vector (inferior direction)
ZAXIS	Coordinate system: z unit vector (modiolar axis)

## 2 Experimental design, materials, and methods

### 2.1 Background and summary

The human inner ear is an organ with remarkable morphological complexity. The membranous structures and fluid compartments involved for the hearing and balance senses are encapsulated in a void, the bony labyrinth, embedded within the densest bone of the human body. In contrast to the microscopic structures of the inner ear, which to date can only be effectively visualized by means of postmortem imaging, the bony labyrinth is sufficiently well visible in clinical computed-tomography (CT) images with sub-millimeter resolution. The particular shape of the bony labyrinth's portions, the cochlea, the vestibule and the semicircular canals, are of interest in several scientific and clinical areas. While the semicircular canals provide a perfect configuration to capture angular acceleration and deceleration of the head [1], it is still unclear why the auditory part of the inner ear, the cochlea, exhibits its coiled appearance. Most evidence suggests that the cochlea has its spiral shape due to spatial restrictions [2,3], but physiological benefits such as improved low frequency hearing are also considered influential [4]. In the human embryo, the inner ear begins to form out of the otic vesicle 5 weeks after fertilization, differentiating and expanding to already reach its near adult size until about 18 weeks [5]. Because of its prenatal formation and preservation within the skull, the bony labyrinth is considered to be insulated from environmental factors and therefore of particular interest for anthropologists. It has been demonstrated that the shape of the bony labyrinth encodes information about the genetic distance from early humans [6] as well as sex-typed differences [7]. A deepened understanding of the morphology is also desired in the fields of otology and neurotology aiming to provide minimally invasive and effective surgical treatment of hearing and balance disorders. An application lies in the design of neuroprostheses, including cochlear [8] and vestibular implants [9]. Moreover, preoperative surgical planning and postoperative radiological outcome evaluation require knowledge of the morphology [10,11]. The data presented herein was generated during a study aimed at extracting the modiolar axis of the cochlea based on intrinsic shape parameters obtained from clinical CT data [12]. To validate the outcome, for each specimen high-resolution micro-CT (uCT) images were acquired and co-registered. Our contribution complements existing data sets such as the Hear-EU multi-scale data set [13] and the micro-slicing based Open-Ear data of 8 specimens [14] by providing a consistent set of images, labels, annotations and surface models for 23 human specimens. Each specimen originates from individual donors. Specimens with cochlear diameters of 8.3–10 mm are contained, covering a wide range of anatomical appearance. The main application of the data set lies in the analysis of inner ear morphology. It is further useful for the validation of partially or fully automated segmentation algorithms to identify the inner ear in clinical CT using the uCT data as a reference.

### 2.2 Specimen preparation

The specimens were received from the Institute of Anatomy, University of Bern, Switzerland. Each donor signed a written informed consent to justify the post-mortem donation and to guarantee that the act of donation meets the legal requirements involved in research with human body parts. Retrieval of the specimens as well as image acquisition and data usage were approved by the local institutional review board (reference number KEK-BE 2016-00887). In total, 23 temporal bone specimens were obtained (14 right and 9 left ear sides). The specimens were obtained either embalmed with Formalin (N = 15) or Thiel (N = 8) [15].

### 2.3 Image acquisition and processing

Image acquisition was performed in two stages. First, clinical CT scans (0.15 × 0.15 × 0.2 mm^3^ voxel size, 94 mA, 120 kV, Somatom Definition Edge, Siemens, Germany) were performed. Then, each specimen underwent high-resolution imaging (0.06 mm isotropic voxel size, 1.5 mA, 68 kV, XtremeCT II Scanco Medical AG, Brüttisellen, Switzerland). The image data was exported using the Digital Imaging and Communications in Medicine (DICOM) standard. Image manipulation and visualization was performed using a proprietary software (AMIRA, version 6.1.1, Thermo Fisher Scientific, Waltham, MA, USA). To avoid the influence of unrelated and movable anatomical regions (e.g. soft tissues and the temporomandibular joints) during image registration, the images were cropped around the bony labyrinth. Registration between CT and uCT images was preformed using the normalized mutual information method available in Amira [16]. Afterwards, the image data were exported as NIFTI (Neuroimaging Informatics Technology Initiative) files.

### 2.4 Labelling and surface generation

The bony labyrinth was manually labelled by an expert in the CT and uCT images. For segmentation a combination of region growing, thresholding and manual correction was used. The image labels were triangulated using a marching cubes surface generation algorithm available in Amira (with smoothing kernel size = 3 voxels) to obtain the 3D surfaces [19].

### 2.5 Landmark annotation and cochlear coordinate system

An experienced otologist annotated 5 anatomical landmarks in two-dimensional CT-views aligned with the cochlear basal turn and the modiolar axis [18]. The landmarks are (i) the center of the round window membrane (RW), the center of the cochlear basal turn (C), the helicotrema (A), the center of the oval window (OW) and the center of the vestibule (V). Using the first three landmarks a cochlear coordinate system was computed according to the consensus reported in Verbist et al. [10]. The landmark C represents the origin of the coordinate system. The positive z axis is obtained by computing and normalizing the vector between landmarks A and C. A temporary x axis is computed as the normalized vector between landmarks RW and C. Then, the vector product of these vectors is computed and normalized (in case of a left ear, the axis is mirrored) to yield the y axis. To ensure orthogonality, the x axis is recomputed as the vector product between the y and z axes.

### 2.6 Code availability

We used a proprietary visualization and analysis software to prepare the data set. However, the data can be loaded and processed with open source and freeware software solutions. For image data visualization and segmentation, the software 3D Slicer was tested (https://www.slicer.org). To visualize and manipulate 3D surface data, the software Meshlab was tested (https://www.meshlab.net). If required, the source code is available through the individual websites.

### 2.7 Technical validation

During data preparation several error sources may influence the outcome, i.e. data labelling. First, cadaver embalming is known to alter characteristics of biological tissue. This is, however, more significant for soft tissue structures. In the case of temporal bones, it has been shown in a previous study that the bony labyrinth is well preserved using either of the fixation methods [17]. Another error source arises from image acquisition, including calibration errors, movement artifacts, metal artifacts and others. The risk of detector system calibration errors is mitigated by routine calibration and maintenance processes at the radiological departments in our clinic, while movement and metal artifacts were not considered relevant with our specimens. To provide optimal image registration, the images were cropped to cover the bony labyrinth, which has distinct features and represents a rigid structure. Each co-registered data set was overlaid and reviewed. The biggest error source is expected during the process of image labelling (segmentation), since many factors, including contrast, windowing and intensity display as well as inter-rater variability have to be taken into account. In the clinical CTs, special care was taken while labelling the round window region, because it sometimes lacks sufficient contrast to clearly identify the round window membrane. For this data set, the segmentation labels were reviewed by an experienced otologists. No anatomical malformations were identified. Finally, surface generation will yield different geometries according to the applied triangulation and filtering parameters. We encourage users of the data set to perform and share further independent labelling sets, which could.
